# HPTLC Method for the Simultaneous Estimation of Valsartan and Hydrochlorothiazide in Tablet Dosage Form

**DOI:** 10.4103/0250-474X.51967

**Published:** 2009

**Authors:** N. J. Shah, B. N. Suhagia, R. R. Shah, N. M. Patel

**Affiliations:** Shri B. M. Shah College of Pharmaceutical Education & Research, Modasa-383 315, India; 1 L. M. College of Pharmacy, Navrangpura, Ahmedabad-380 009, India

**Keywords:** Valsartan, hydrochlorothiazide, simultaneous estimation, HPTLC

## Abstract

A simple, precise, accurate and rapid high performance thin layer chromatographic method has been developed and validated for the simultaneous estimation of valsartan and hydrochlorothiazide in combined dosage forms. The stationary phase used was precoated silica gel 60F_254_. The mobile phase used was a mixture of chloroform: methanol: toluene: glacial acetic acid (6:2:1:0.1 v/v/v/v). The detection of spots were carried out at 260 nm. The method was validated in terms of linearity, accuracy, precision and specificity. The calibration curve was found to be linear between 300 to 800 ng/spot for valsartan and 100 to 600 ng/spot for hydrochlorothiazide. The limit of detection and the limit of quantification for the valsartan were found to be 100 and 300 ng/spot respectively and for hydrochlorothiazide 30 and 100 ng/spot respectively. The proposed method can be successfully used to determine the drug content of marketed formulation.

Valsartan, a nonpeptide, is *N*-(1-oxopentyl)-*N*-[[2'-(1*H*-tetrazol-5-yl) [1,1'-biphenyl]-4-yl] methyl]-L-valine[1]. Hydrochlorothiazide is 6-chloro-3,4-dihydro-2H-1,2,4-benzothiadiazine-7-sulphonamide,1,1-dioxide. In patients with moderate hypertension, first-line therapy with the fixed-dose of valsartan/hydrochlorothiazide combination leads to BP normalization with high response rates. Literature survey revealed that a few HPLC and spectrophotometry methods are reported for the estimation of valsartan in biological samples such as plasma[2–7]. So far no HPTLC method has been reported for the estimation of valsartan and hydrochlorothiazide in combined dosage forms. In the present investigation, an accurate and precise HPTLC method for the simultaneous estimation of valsartan and hydrochlorothiazide in combined dosage forms has been developed.

Valsartan and hydrochlorothiazide standard were procured as a gift sample from Jubilant Organosys Ltd., Mysore, India. Silica gel 60F_254_ TLC plates (10×10 cm, layer thickness 0.2 mm, E. Merck, Mumbai, India) were used as a stationary phase. All chemicals and reagents used were of analytical grade. Tablets containing valsartan (80 mg) and hydrochlorothiazide (12.5 mg) were procured from the local pharmacy (Valzarr-H, Torrent Pharmaceutical Ltd, Ahmedabad, India). A Camag HPTLC system comprising of Camag Linnomate V automatic sample applicator, Hamilton syringe (100 μl), Camag TLC Scanner 3, Camag WinCATS software, Camag Twin-trough chamber (10×10 cm) and ultrasonicator were used during study.

Valsartan and hydrochlorothiazide (25 mg) each were weighed accurately, dissolved and diluted with methanol to obtain the final concentration of 100 μg/ml of each drug. Twenty tablets were weighed accurately and ground to a fine powder. Weight equivalent to 25 mg of valsartan and hydrochlorothiazide were transferred to conical flask and mixed with methanol. The solution was sonicated for 15 min. The extracts were filtered through Whatman filter paper No. 41 and residue was washed thoroughly with methanol. The extracts and washing were pooled and transferred to a 250 ml volumetric flask and volume was made up to 250 ml with methanol. Required dilutions were made to get 100 μg/ml of valsartan and hydrochlorothiazide.

TLC plates were prewashed with methanol. Activation of plates was done in an oven at 50° for 5 min. The chromatographic conditions maintained were precoated silica gel 60F_254_ aluminum sheets (10×10 cm) as stationary phase, chloroform:methanol:toluene:glacial acetic acid (6:2:1:0.1 v/v/v/v) as mobile phase, chamber and plate saturation time of 30 min, migration distance allowed was 72 mm, wavelength scanning was done at 260 nm keeping the slit dimensions at 5×0.45 mm. A deuterium lamp provided the source of radiation. Standard solutions of valsartan and hydrochlorothiazide were spotted and developed. Photometric measurements were performed at 260 nm in reflectance mode with Camag TLC scanner 3 using WinCATS software.

Aliquots of 3.0, 4.0, 5.0, 6.0, 7.0, 8.0 μl of standard solution of valsartan and 1.0, 2.0, 3.0, 4.0, 5.0, 6.0 μl of hydrochlorothiazide were applied on the TLC plate (100 μg/ml of drug). TLC plate was dried, developed and analyzed photometrically as described earlier. The standard calibration curve was generated using regression analysis with Microsoft excel. Sample solutions of the marketed formulation were spotted on to the same plate followed by development scanning. The analysis was repeated in triplicate. The content of the drug was calculated from the peak areas recorded. The developed method was validated in terms of linearity, accuracy, limit of detection, limit of quantification, intra-day and inter-day precision and repeatability of measurement as well as repeatability of sample application[8].

A solvent system that would give dense and compact spots with significant R_f_ values were desired for quantification of valsartan and hydrochlorothiazide in pharmaceutical formulations. The mobile phase consisting of chloroform:methanol:toluene:glacial acetic acid (6:2:1:0.1 v/v/v/v) gave R_f_ values of 0.36±0.04 and 0.63±0.03 for valsartan and hydrochlorothiazide, respectively (fig. 1). The linear regression data (n=5, Table 1) showed a good linear relationship over a concentration range of 300-800 ng/spot and 100-600 ng/spot for valsartan and hydrochlorothiazide, respectively. The limit of detection and limit of quantification for valsartan was found to be 100 and 300 ng/spot and for hydrochlorothiazide, 30 and 100 ng/spot, respectively.

**Fig. 1:**
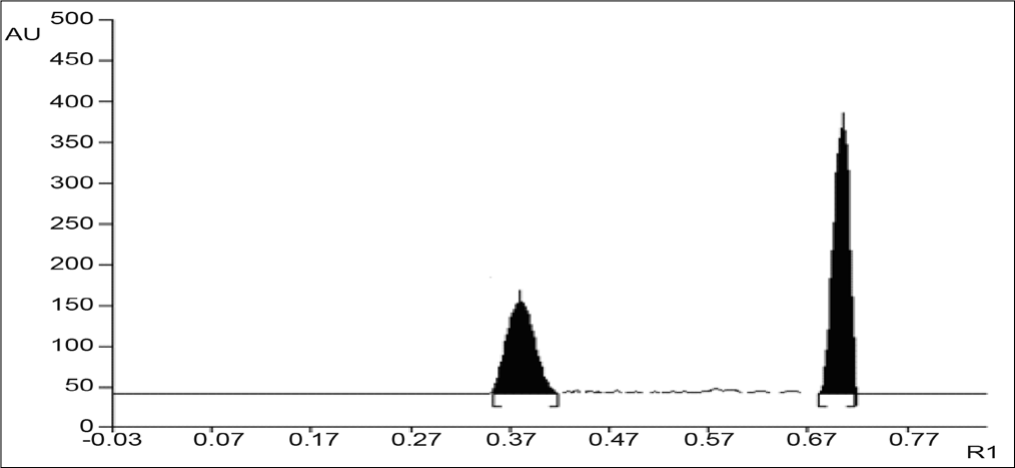
A typical HPTLC chromatogram of valsartan and hydrochlorothiazide.

**TABLE 1:** METHOD VALIDATION PARAMETERS OF PROPOSED METHOD

Parameters	Values	
	
	Valsartan	Hydrochlorothiazide
Linearity range (ng/spot)	300-800	100-600
Correlation coefficient (r)	0.9961	0.998
Regression equation (y=mx+c)		
Slope (m)	3.0779	4.1895
Intercept (c)	43.295	2.56
Limit of detection (LOD)	100 ng/spot	30 ng/spot
Limit of quantification (LOQ)	300 ng/spot	100 ng/spot
Precision (% CV)		
Repeatability of application (n=5)	0.98	0.83
Repeatability of measurement (n=5)	0.43	0.49

The intra-day precision was determined by analyzing standard solutions in the concentration range of 400 ng/spot to 700 ng/spot for valsartan and 200 ng/spot to 500 ng/spot for hydrochlorothiazide for 3 times on the same day while inter-day precision was determined by analyzing corresponding standards daily for 3 day over a period of one week. The intra-day and inter-day coefficients of variation for both drugs were found to be in the range of 0.27-1.19% and 0.12-1.05%, respectively. These values indicate that the method is precise.

Repeatability of sample application was assessed by spotting 4 μl of drug solution 5 times on a TLC plate followed by development of plate and recording the peak area for 5 spots. The % RSD for peak area values of valsartan and hydrochlorothiazide were found to be 0.98 and 0.83, respectively. Repeatability of measurement of peak area was determined by spotting 4 μl of valsartan and hydrochlorothiazide solution on a TLC plate and developing the plate. The separated spot was scanned five times without changing the position of the plate and % RSD for measurement of peak area of valsartan and hydrochlorothiazide were found to be 0.43 and 0.49, respectively. To confirm the specificity of the proposed method, the solution of the formulation was spotted on the TLC plate, developed and scanned. It was observed that the excipients present in the formulation did not interfere with the peaks of valsartan and hydrochlorothiazide.

Recovery studies of the drugs were carried out for the accuracy parameter. These studies were carried out at three levels i.e. multiple level recovery studies. Sample stock solutions from tablet formulation of 100 μg/ml were prepared. Dilutions were made and recovery studies were performed. Percentage recovery was found to be within the limits as listed in Table 2. The assay value for the marketed formulation was found to be within the limits as listed in Table 2. The low RSD value indicated the suitability of the method for routine analysis of valsartan and hydrochlorothiazide in pharmaceutical dosage forms.

**TABLE 2:** ASSAY AND RECOVERY STUDIES OF VALSARTAN AND HYDROCHLOROTHIAZIDE

Brand name	Label Claim mg / tablet	Total Amount added (mg)	Amount recovered* (mg) ± SD	% Recovery ± SD	% Assay*
Valzarr-H	Valsartan 80	40	39.83± 0.98	99.59±0.98	
		80	79.28±1.50	99.10±1.50	100.78±0.43
		120	120.70±0.79	100.59±0.79	
	Hydrochlorothiazide	6.25	6.27±1.69	100.87±1.69	
	12.5	12.50	12.32±0.95	99.92±0.95	99.81±0.85
		18.75	19.13±0.87	102.06±0.87	

* Each value is a mean ± standard deviation of three determinations. Valzarr-H is a brand of Torrent Pharmaceutical Ltd.

The developed HPTLC technique is simple, precise, specific and accurate and the statistical analysis proved that method is reproducible and selective for the analysis of valsartan and hydrochlorothiazide in bulk drug and tablet formulations.
